# Hypercholesterolemia affects cardiac function, infarct size and inflammation in APOE*3-Leiden mice following myocardial ischemia-reperfusion injury

**DOI:** 10.1371/journal.pone.0217582

**Published:** 2019-06-14

**Authors:** Niek J. Pluijmert, Melina C. den Haan, Vanessa L. van Zuylen, Paul Steendijk, Hetty C. de Boer, Anton J. van Zonneveld, Willem E. Fibbe, Martin J. Schalij, Paul H. A. Quax, Douwe E. Atsma

**Affiliations:** 1 Department of Cardiology, Leiden University Medical Center, Leiden, The Netherlands; 2 Department of Nephrology, Leiden University Medical Center, Leiden, The Netherlands; 3 Department of Immunohematology and Blood Transfusion, Leiden University Medical Center, Leiden, The Netherlands; 4 Department of Surgery, Leiden University Medical Center, Leiden, The Netherlands; 5 Einthoven Laboratory for Experimental Vascular Medicine, Leiden University Medical Center, Leiden, The Netherlands; Scuola Superiore Sant'Anna, ITALY

## Abstract

**Background:**

Hypercholesterolemia is a major risk factor for ischemic heart disease including acute myocardial infarction. However, long-term effects of hypercholesterolemia in a rodent myocardial ischemia-reperfusion injury model are unknown. Therefore, the effects of diet-induced hypercholesterolemia on cardiac function and remodeling were investigated up to eight weeks after myocardial ischemia-reperfusion (MI-R) injury which was induced in either normocholesterolemic (NC-MI) or hypercholesterolemic (HC-MI) APOE*3-Leiden mice.

**Methods:**

Left ventricular (LV) dimensions were serially assessed using parasternal long-axis echocardiography followed by LV pressure-volume measurements. Subsequently, infarct size and the inflammatory response were analyzed by histology and fluorescence-activated cell sorting (FACS) analysis.

**Results:**

Intrinsic LV function eight weeks after MI-R was significantly impaired in HC-MI compared to NC-MI mice as assessed by end-systolic pressure, dP/dt_MAX_, and -dP/dt_MIN_. Paradoxically, infarct size was significantly decreased in HC-MI compared to NC-MI mice, accompanied by an increased wall thickness. Hypercholesterolemia caused a pre-ischemic peripheral monocytosis, in particular of Ly-6C^hi^ monocytes whereas accumulation of macrophages in the ischemic-reperfused myocardium of HC-MI mice was decreased.

**Conclusion:**

Diet-induced hypercholesterolemia caused impaired LV function eight weeks after MI-R injury despite a reduced post-ischemic infarct size. This was preceded by a pre-ischemic peripheral monocytosis, while there was a suppressed accumulation of inflammatory cells in the ischemic-reperfused myocardium after eight weeks. This experimental model using hypercholesterolemic APOE*3-Leiden mice exposed to MI-R seems suitable to study novel cardioprotective therapies in a more clinically relevant animal model.

## Introduction

Hypercholesterolemia plays an important role in the occurrence of atherosclerosis [1] and is a major risk factor for ischemic heart disease [2]. Several clinical studies demonstrated an adverse effect of hypercholesterolemia on coronary heart disease events and left ventricular (LV) systolic function after suffering a myocardial infarction (MI) [3,4] and reported positive effects of lipid lowering therapies [5,6].

Experimental MI studies have reported controversial findings regarding the effect of diet-induced hypercholesterolemia on cardiac function following myocardial-ischemia reperfusion (MI-R). Acute cholesterol feeding, up to three weeks, is associated with increased MI-R injury in animals [7,8]. Also, prolonged exposure to diet-induced hypercholesterolemia, during up to 20 weeks, followed by MI-R injury with reperfusion periods up to 24 hours showed a reduced hemodynamic performance [9] and a negative inotropic effect in animals [10]. In addition, myocardial injury was exacerbated by increased cardiomyocyte apoptosis [11], upregulation of the myeloperoxidase-related inflammatory response [12,13], reduced myocardial nitric oxide synthesis [14], and increased myocardial necrosis [15]. Conversely, other studies reported hypercholesterolemia to cause an improved mechanical recovery [9,10], a positive inotropic effect [16], and decreased cardiac necrosis [17]. However, information on follow-up periods longer than 24 hours is lacking.

To realistically study the effects of hypercholesterolemia on cardiovascular outcome after MI, animal models should mimic the clinical setting of expedited reperfusion therapy upon acute coronary artery occlusion. This was also underscored by a recent position paper of the ESC which emphasizes the importance of studying novel cardioprotective therapies in animal models more closely mimicking the clinical situation in order to improve final successful translation to the clinical setting [18]. In this perspective, the APOE*3-Leiden transgenic mouse seems a particularly appropriate animal model [19]. APOE*3-Leiden mice develop advanced aortic atherosclerotic lesions resembling their human counterparts when exposed to cholesterol feeding [20,21] and proved to be useful in studying the environmental and genetic factors in the occurrence of hyperlipidemia [21,22] and the development of atherosclerosis [21,23]. In addition, APOE*3-Leiden mice have been used to study the effects of various lipid lowering therapies [24–26]. Although APOE*3-Leiden mice differ from the human situation, because of the absence of coronary atherosclerosis resulting in coronary plaque formation and the lack of rupture followed by thrombus formation, it does provide an excellent model to study the effects of hypercholesterolemia on the pathophysiological processes in an animal model after surgical interventions [27–30] including induction of MI. Furthermore, hypercholesterolemia-induced atherosclerosis itself is considered to be an inflammatory disease which contributes to and affects the post-ischemic inflammatory response [1,31].

The aim of the present study was to investigate the long-term effects of hypercholesterolemia on MI-R induced injury in APOE*3-Leiden mice, concomitantly studying the effectivity and reproducibility of a small animal model more closely mimicking the clinical situation. Therefore, we employed a follow-up period of eight weeks after MI-R, focusing on cardiac function, infarct size, and the post-ischemic inflammatory response.

## Materials and methods

A schematic overview of the complete study protocol shown as a timeline can be found as a supplemental figure online (S1 Fig).

### Animals and diets

Transgenic female APOE*3-Leiden mice [19], backcrossed for more than 40 generations on a C57Bl/6J background, aged 10–12 weeks at the start of a dietary run-in period (bred in the animal facility of the Leiden University Medical Center), were used for this experiment. Mice were randomly assigned to either a normal chow (normocholesterolemic, NC) or a semisynthetic Western-type diet supplemented with 0.4% cholesterol (hypercholesterolemic, HC) (AB Diets, Woerden, The Netherlands). Female rather than male APOE*3-Leiden mice were used because of their higher and stable plasma cholesterol and triglyceride levels, confined to the VLDL/LDL-sized lipoprotein fraction [32,33]. The diet was started four weeks prior to surgery, earlier proven to attain a stable hypercholesterolemic phenotype, and was continued until the end of the experiment. Mice were housed under standard conditions in conventional cages and received food and water ad libitum. All animal experiments were approved by the Institutional Committee for Animal Welfare of the Leiden University Medical Center (approval reference number 09131) and conformed to the *Guide for the Care and Use of Laboratory Animals* (NIH Publication No. 85–23, revised 2011). All surgery was performed under isoflurane anesthesia, and all efforts were made to minimize suffering by using buprenorfine analgesia.

### Plasma lipid analysis

Plasma total cholesterol (TC) and triglyceride (TG) levels were determined prior to diet exposure, before induction of MI, and four and eight weeks after surgery. After a 4-hour fasting period, blood was obtained via tail vein bleeding (~50μl) and assayed for plasma total cholesterol (TC) and triglyceride (TG) levels using commercially available enzymatic kits according to the manufacturer’s protocols (11489232 and 11488872; Roche Diagnostics, Mannheim, Germany, respectively)

### Surgical myocardial ischemia-reperfusion protocol

Surgical ligation of the left anterior descending (LAD) coronary artery followed by permanent reperfusion was performed at day 0 in 12–14 weeks old female APOE*3-Leiden mice as described previously [34]. Briefly, mice were pre-anesthetized with 5% isoflurane in a gas mixture of oxygen and room air and placed in a supine position on a heating pad (37°C). After endotracheal intubation and ventilation (rate 160 breaths/min, stroke volume 190μl; Harvard Apparatus, Holliston, MA, USA), mice were kept anesthetized with 2% isoflurane during approximately 60 minutes. Subsequently, a left thoracotomy was performed in the 4^th^ intercostal space and the LAD coronary artery was ligated using a 7–0 prolene suture. A knot was tied on a 1mm section of a plastic tube placed on top of the LAD to occlude the coronary artery for 45 minutes. Ischemia was confirmed by myocardial blanching and ECG changes. Muscle flaps were folded back, covered with a pre-warmed wet surgical mesh, and body temperature was kept constant between 35–37°C during this period. After 35 minutes of ischemia mice received an intraperitoneal injection of lidocaine (6mg/kg) [35] to prevent cardiac arrhythmias caused by reperfusion. After 45 minutes of ischemia permanent reperfusion was established. The thorax was closed in layers with 5–0 prolene suture and mice were allowed to recover. Analgesia was obtained with buprenorfine s.c. (0.1mg/kg) pre-operative and 12h post-operative. The experimental protocol consisted of a normal diet MI-R group (NC-MI, n = 16) and cholesterol-enriched diet MI-R group (HC-MI, n = 18).

### Echocardiography

To evaluate LV function, *in vivo* transthoracic echocardiography was performed in anesthetized (2% isoflurane) mice using a 15-45MHz RMV707B probe interfaced with a Vevo 770 imaging system (VisualSonics Inc, Toronto, Canada). Two-dimensional echocardiography was achieved in all mice before induction of MI (week 0) to assess baseline cardiac function and serve as an internal control. Subsequently, LV function was measured at one, three, and eight weeks after MI-R during circa 10 minutes per analysis. Mice were placed on a heating table in a supine position, with their extremities fixed to four electrocardiography leads. The chest was shaved to minimize ultrasound attenuation and warmed Aquasonic gel (Parker Laboratories Inc, Fairfield, USA) was applied to optimize visibility.

Parasternal long-axis B-Mode, M-Mode, and EKG-gated Kilohertz Visualization (EKV) images were obtained with appropriate angulation and acquisition of maximum LV length, from apex to aortic valve. Datasets were analyzed in a blinded manner, using Visual Sonics software version 3.0.0 (2008). After tracing the end-systolic and end-diastolic endocardial LV area of parasternal long-axis EKV images [36] LV end-diastolic volume (EDV), LV end-systolic volume (ESV), LV ejection fraction (EF), and cardiac output (CO) were calculated.

### Hemodynamic measurements

After eight weeks, hemodynamics and LV function indices were determined by invasive LV pressure-volume (PV) relationships. After induction of anesthesia (2% isoflurane) a midline neck incision was performed, and a 1.2F PV catheter (FTS-1212B-4518; Scisense Inc, Ontario, Canada) connected to an ADV signal processor (Scisense Inc) was inserted via the right carotid artery and positioned optimally into the LV to generate high-fidelity PV signals. On-line display and acquisition of the signals (2000 samples·s-1) was obtained with a PowerLab 8/30 system and LabChart Pro software (AD Instruments GmbH, Spechbach, Germany). Parallel conductance was obtained with the hypertonic saline method using intravenous bolus injections of ~5μl 10% saline [37] and calibrated with corresponding echocardiographic values of CO. Total execution of hemodynamic measurements took about 25 minutes. All data were analyzed off-line in a blinded fashion with custom-made software.

PV signals were obtained in steady state to measure heart rate (HR), CO, ESV, EDV, end-systolic and end-diastolic pressure (ESP and EDP), maximal and minimal rates of LV pressure change (dP/dt_MAX_ and dP/dt_MIN_), isovolumetric relaxation time constant (Tau), stroke work (SW), effective arterial elastance (E_A_), end-systolic peak isovolumic pressure (ESP_iso_), end-systolic elastance (E_ES_), end-systolic intercept volume (ESV_int_), end-diastolic stiffness (E_ED_), and end-diastolic intercept volume (EDV_int_).

After measurements, the heart and lungs were quickly excised. Hearts were immersion-fixated in 4% paraformaldehyde for 24 hours and embedded in paraffin. The body weight and wet lung weight were measured from all animals and lungs were then freeze-dried. The difference between wet and dry lung-weight was used as a measure of pulmonary congestion.

### Infarct size, LV wall thickness and vascular density

Paraffin-embedded hearts were cut into serial transverse sections of 5μm along the entire long-axis of the LV and subsequently mounted on slides (n = 8 for each group). To analyze collagen deposition as an indicator of the fibrotic area, every 50^th^ section of each heart was stained with Sirius Red resulting in approximately 15 stained sections of each heart. Infarct size was determined by planimetric measurement of all sections and calculated as fibrotic area divided by the total LV wall surface area including the interventricular septum. LV wall thickness was measured in five different sections equally distributed through the infarcted area. Per section, wall thickness was analyzed in the mid-infarcted area, both border zones, and interventricular septum. Measurements were performed perpendicular to the ventricular or septal wall.

To determine the vascular profile, serial sections were stained for PECAM-1 (CD31, clone MEC13.3, 550274; BD Pharmingen, San Diego, CA, USA). Subsequent to incubations with an appropriate biotinylated secondary antibody and the signal amplifying ABC system (Vectastain; Vector Laboratories, Burlingame, CA, USA), the reaction product was visualized with 3,3^‘^-diaminobenzidine and counterstained with Mayer’s hematoxillin. Vascular density was determined by quantifying the number of PECAM-1 positive blood vessels per 0.25mm^2^ per section, differentiating between small (<20μm) and large (>20μm) vessels, in the infarcted border zones (4 areas), and infarcted myocardium (5 areas). All measurements were performed by an observer blinded to the groups, using the Image-Pro Plus software package (Media Cybernetics Inc, Bethesda, MD, USA).

### Inflammatory response

To study the *in vivo* effects of the cholesterol-enriched diet and MI-R injury, whole blood was analyzed for peripheral monocytosis one week before induction of MI (NC-MI, n = 11, and HC-MI, n = 15) and three weeks after MI-R (NC-MI, n = 6, and HC-MI, n = 7). Hematological values obtained were white blood cell counts (WBC, x10^6^/ml), red blood cell counts (RBC, x10^9^/ml), and platelets (PLT, x10^6^/ml) using a semi-automatic hematology analyzer F-820 (Sysmex; Sysmex Corporation, Etten-Leur, The Netherlands). For FACS analysis, 35μl of whole blood was incubated for 30 minutes on ice with directly conjugated antibodies directed against Ly-6C-FITC (AbD Serotec, Dusseldorf, Germany), Ly-6G-PE (BD Pharmingen, San Diego, CA, USA), CD11b-APC (BD Pharmingen, San Diego, CA, USA), CD115-PerCP (R&D Systems, Minneapolis, MN, USA), and CD45R-APC-Cy7 (eBioscience, San Diego, CA, USA). Monocytes were gated based on their expression profile: Ly-6G-negative, CD11b-positive, and CD115-positive. Pro-inflammatory monocytes were identified based on high Ly-6C expression levels.

For analysis of the local cardiac inflammatory response eight weeks after MI-R, paraffin sections of the mid-infarct region of the heart were stained using antibodies against leukocytes (anti-CD45, 550539; BD Pharmingen, San Diego, CA, USA) and macrophages (anti-Mac-3, 550292; BD Pharmingen, San Diego, CA, USA). The number of leukocytes and macrophages were expressed as a number per 0.25mm^2^ in the septum (2 areas), border zones (2 areas), and infarcted myocardium (5 areas).

### Statistical analysis

Values were expressed as means ± SEM. Comparisons of parameters between the NC-MI and HC-MI groups were made using independent samples *t*-test or 2-way analysis of variance with repeated measures and Bonferroni’s posttest in case of multiple time points. A value of *P*<0.05 was considered to represent a significant difference. All statistical procedures were performed using SPSS 23.0.0 (SPSS Inc–IBM, Armonk, NY, USA) and GraphPad Prism 6.02 (GraphPad Software Inc, La Jolla, CA, USA).

## Results

### Plasma lipid profiles and animal characteristics

**Fig 1:**
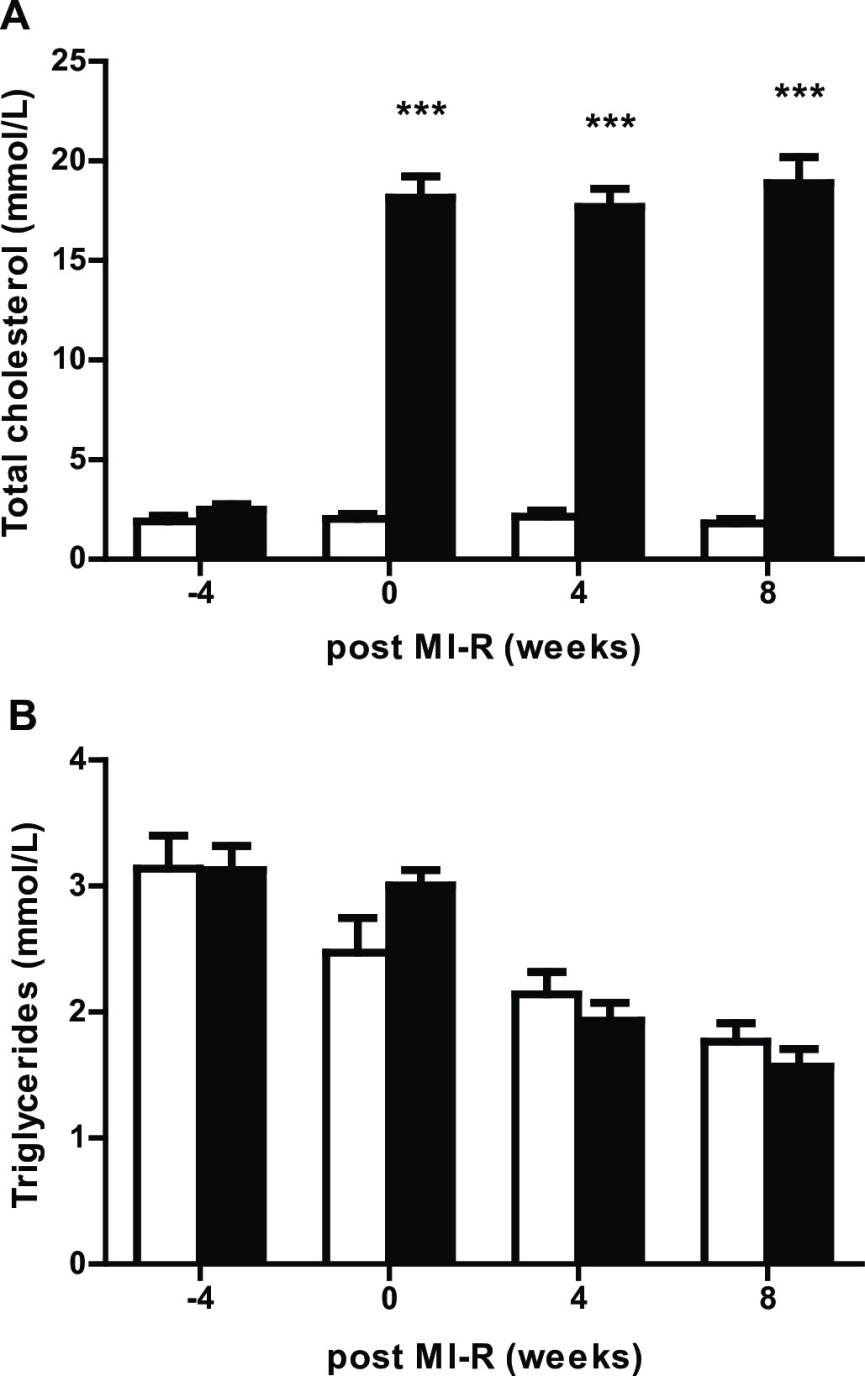
Lipid profiles. Plasma (A) total cholesterol and (B) triglycerides levels in the NC-MI (open bars) and HC-MI (closed bars) group. Data are means ± SEM. ***P<0.001 vs. NC-MI group.

Total cholesterol plasma levels in the HC-MI group were increased after exposure to the cholesterol-enriched diet for four weeks compared to the normal chow diet group (18.2±1.1mmol/L vs. 2.0±0.3mmol/L, *P*<0.001) and remained stable during the experimental protocol. Triglycerides levels (3.0±0.1mmol/L vs. 2.5±0.3mmol/L) were not significantly different between groups (Fig 1).

No difference in body weight (BW) was observed between groups prior to the induction of MI-R. However, eight weeks after MI-R weight gain as expressed by BW change was increased in HC-MI as compared to NC-MI mice. No difference in the amount of lung fluid was observed between both groups (Table 1). In addition, surgical survival rates were 84.0% in the HC-MI vs. 79.2% in the NC-MI group and 85.7% vs. 84.2% respectively during subsequent follow-up period of 8 weeks.

**Table 1:** Animal characteristics. Body weight (BW), heart weight (HW). Values are means ± SEM. **P<0.01 vs. NC-MI.

		Normal	Cholesterol
	T (wk)	NC-MI	HC-MI
N		16	18
**age** (days)	0	86 ± 3	88 ± 2
**BW** (g)	0	20.9 ± 0.3	20.5 ± 0.4
	8	22.4 ± 0.4	22.9 ± 0.4
**BW change** (%)		7.3 ± 1.3	12.1 ± 1.1**
**HW** (mg)	8	191 ± 16	176 ± 5
**HW/BW ratio** (mg/g)		8.2 ± 0.6	7.5 ± 0.2
**lung fluid** (mg)		208 ± 11	205 ± 11

### Echocardiography

**Fig 2:**
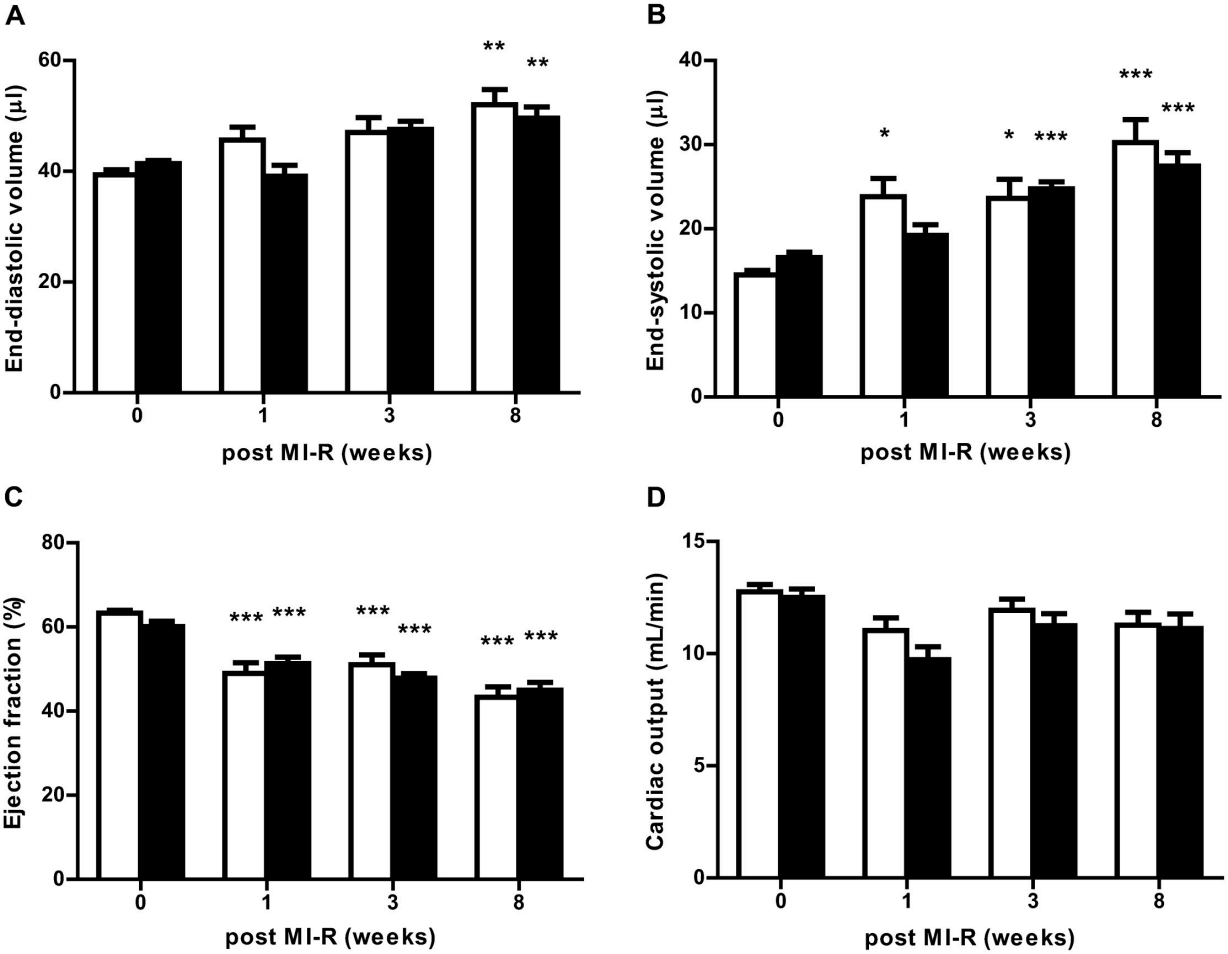
Serial echocardiography. (A) End-diastolic volume, (B) end-systolic volume, (C) ejection fraction, and (D) cardiac output in NC-MI (open bars) and HC-MI (closed bars) groups at baseline, 1, 3, and 8 weeks after MI-R (n = 16–18). Data are means ± SEM. *P<0.05, **P<0.01, ***P<0.001 all vs. week 0.

Serial echocardiography eight weeks post MI-R revealed an increase in LV dimensions in both groups as compared to baseline cardiac function before MI-R. In the HC-MI group EDV (49.6±2.1μl vs. 41.4±0.5μl, *P*<0.001) and ESV (27.4±1.7μl vs. 16.6±0.6μl, *P*<0.001) were increased. In the NC-MI group the EDV (52.0±2.7μl vs. 39.4±0.9μl, *P*<0.001) and ESV (30.3±2.7μl vs. 14.5±0.5μl, *P*<0.001) were increased as well (Fig 2A and 2B). This was accompanied by a progressive impairment of LV function eight weeks after MI-R as indicated by a decrease in EF (Fig 2C) in the HC-MI (45.0±2.0% vs. 60.1±1.3% before MI-R, *P*<0.001) and NC-MI group (43.3±2.5% vs. 63.3±0.7% before MI-R, *P*<0.001). There were no differences observed between the HC-MI and NC-MI group during the experiment.

### Hemodynamic measurements

**Fig 3:**
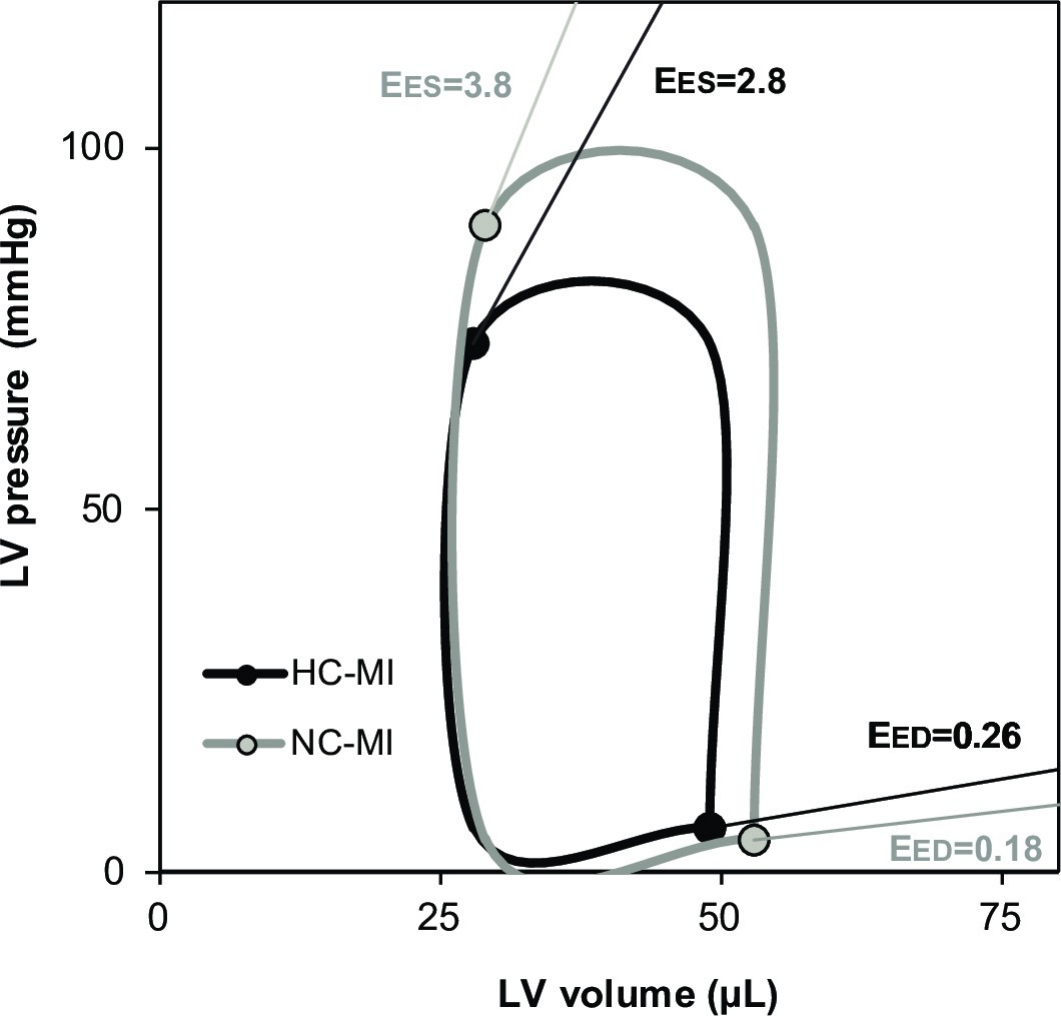
Pressure-volume loops. PV loops 8 weeks after MI-R of the NC-MI and HC-MI groups. The oblique lines represent the end-systolic (E_ES_) and end-diastolic (E_ED_) pressure-volume relations.

The functional PV loop-derived data of the groups are presented in Table 2. In accordance with the echocardiographic data, LV volumes did not differ between the HC-MI and NC-MI group. However, when compared to the NC-MI group, a marked impaired intrinsic LV function was found in the HC-MI group as demonstrated by a significantly depressed ESP, dP/dt_MAX_, and -dP/dt_MIN_. Summarized schematic PV loops are demonstrated in Fig 3.

**Table 2:** Pressure-volume loops-derived LV function indices.

	NC-MI	HC-MI
**HR** (beats min^-1^)	551 ± 18	531 ± 20
**CO** (ml min^-1^)	13.5 ± 2.5	11.4 ± 1.0
**ESV** (μl)	29 ± 5	28 ± 5
**EDV** (μl)	53 ± 7	49 ± 6
**ESP** (mmHg)	89 ± 2	73 ± 3***
**EDP** (mmHg)	4.4 ± 0.6	5.9 ± 0.8
**dP/dt**_**MAX**_ (mmHg ms^-1^)	8.3 ± 0.4	6.1 ± 0.4***
**-dP/dt**_**MIN**_ (mmHg ms^-1^)	6.8 ± 0.2	5.3 ± 0.4**
**Tau** (ms)	9.8 ± 0.3	11.1 ± 0.8
**SW** (mmHg.ml)	1.9 ± 0.2	1.6 ± 0.0
**E**_**A**_	4.8 ± 1.1	3.7 ± 0.3
**ESP**_**iso**_	164 ± 5	131 ± 5***
**ESPVR**		
**slope: E**_**ES**_ (mmHg μl^-1^)	3.8 ± 0.6	2.8 ± 0.3
**intercept: ESV**_**int**_ (μl)	26.3 ± 4.9	29.9 ± 5.1
**EDPVR**		
**slope: E**_**ED**_ (mmHg μl^-1^)	0.18 ± 0.03	0.26 ± 0.05
**intercept: EDV**_**int**_ (μl)	61.1 ± 10.9	46.7 ± 4.0

CO, cardiac output; dP/dt_MAX_, maximum rate of pressure increase; -dP/dt_MIN_, maximum rate of pressure decrease; E_A_, effective arterial elastance; EDP, end-diastolic pressure; EDPVR, end-diastolic pressure-volume relationship; EDV, end-diastolic volume; EDV_int_, end-diastolic intercept volume; E_ED_, end-diastolic stiffness; E_ES_, end-systolic elastance; ESP, end-systolic pressure; ESP_iso_, end-systolic peak isovolumic pressure; ESPVR, end-systolic pressure-volume relationship; ESV, end-systolic volume; ESV_int_, end-systolic intercept volume; HR, heart rate; SW, stroke work; Tau, relaxation time constant. Values are means ± SEM.

**P<0.01

***P<0.001 vs. NC-MI.

### Infarct size, LV wall thickness, and vascular density

**Fig 4:**
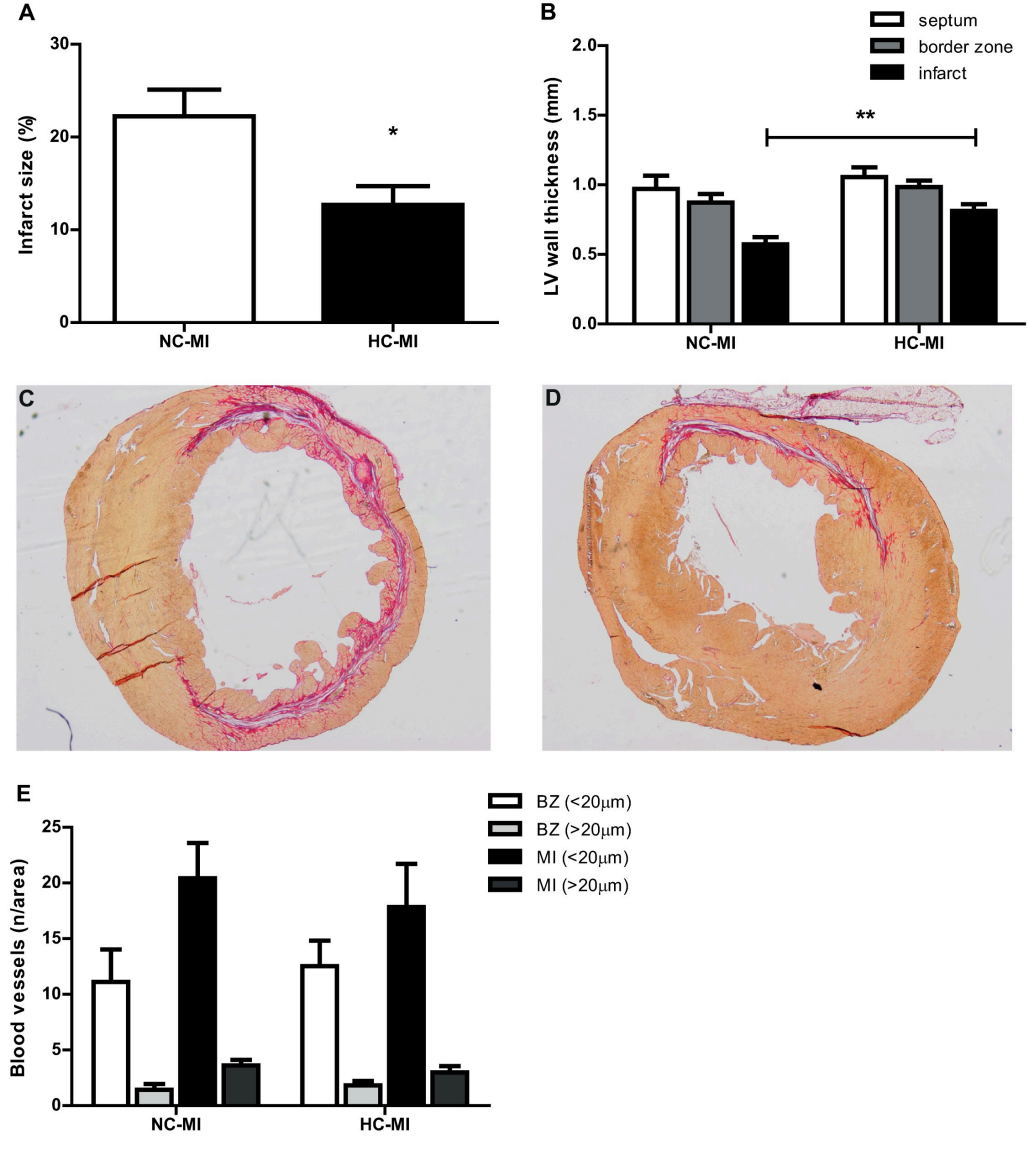
Infarct size, LV wall thickness, and vascular profile 8 weeks after MI-R. Infarct size (n = 8) was significantly smaller in the HC-MI group compared with the NC-MI group (A). Wall thickness of the infarct area was significantly larger in the HC-MI group (B). Sirius red staining in the HC-MI group showed a more pronounced subendocardial and epicardial border of surviving cardiomyocytes in the HC-MI group (D) compared to the NC-MI group (C). Vascular analysis showed no differences in number of small capillaries (<20μm) or large vessels (>20μm) in the border zone (BZ) or infarct area (MI) between both groups (E). Data are means ± SEM. *P<0.05, **P<0.01.

Histological evaluation eight weeks after MI-R showed a smaller infarct area in the HC-MI group compared to the NC-MI group (12.7±2.0% vs. 22.2±2.9%, *P* = 0.017, Fig 4A). The subendocardial and epicardial surviving borders of cardiomyocytes in the HC-MI group (Fig 4D) were larger compared with the NC-MI group (Fig 4C). This resulted in an increased LV wall thickness in the mid-infarct area in the HC-MI group as compared to the NC-MI group (0.81±0.05mm vs. 0.57±0.05mm, *P* = 0.007, Fig 4B).

Analysis of the vascular profile in HC-MI and NC-MI groups showed no significant differences in the number of small capillaries (<20μm) and large vessels (>20μm) between the groups (Fig 4E).

### Inflammatory response

**Fig 5:**
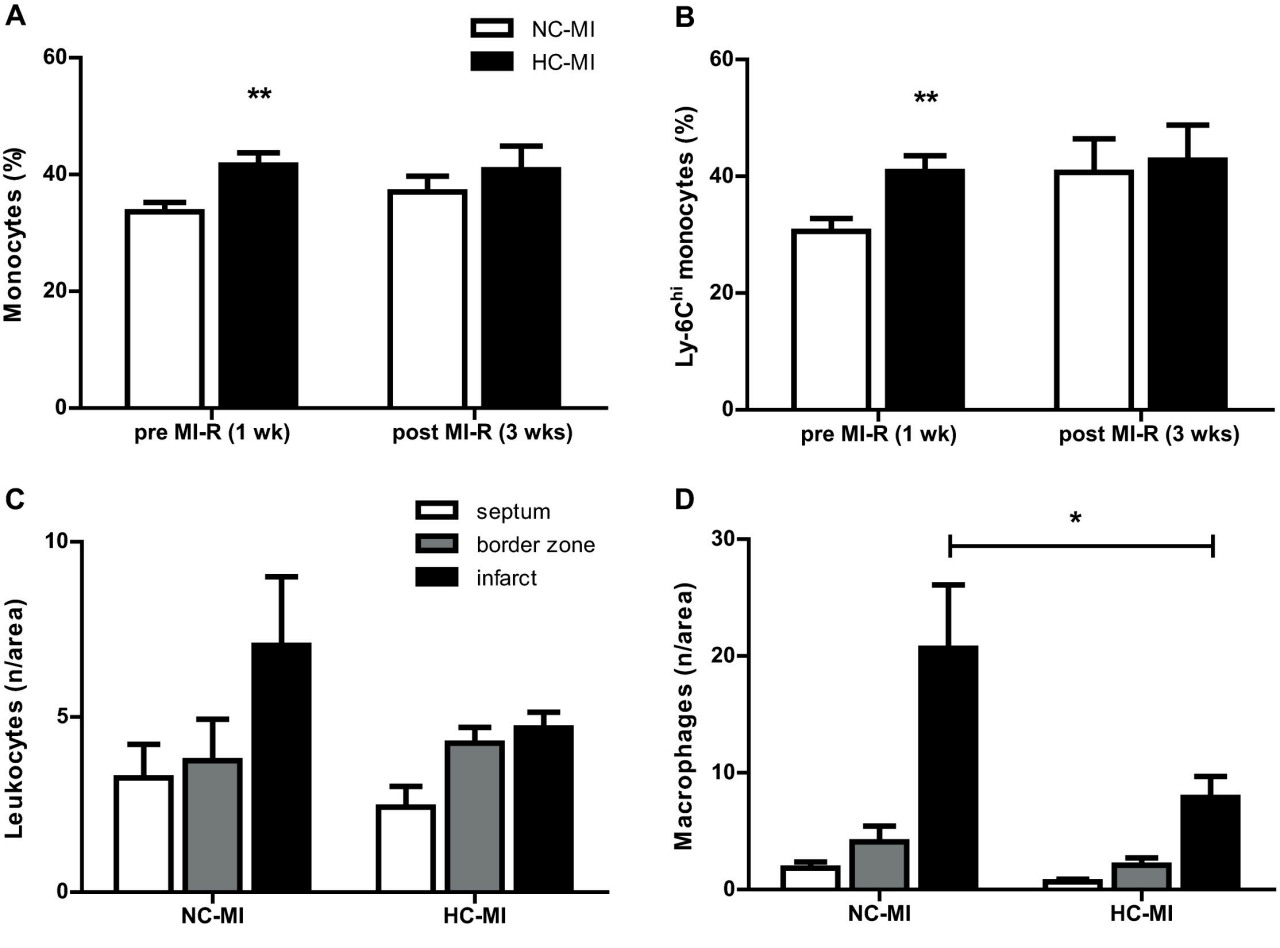
Inflammatory response as a result of the cholesterol-enriched diet and MI-R. Quantification of monocytes as a percentage of total leukocytes (A) and activated (Ly-6C^hi^) monocytes as a percentage of total monocytes (B) in whole blood 1 week before induction of MI-R (n = 11–15) and 3 weeks after MI-R (n = 6–7). **P<0.01 vs. time-corresponding NC-MI group. Quantification of leukocytes (C) and macrophages (D) 8 weeks after MI-R in the infarct area, border zone and septum, expressed as the number per area. Data are means ± SEM. *P<0.05.

After a dietary run-in period, the HC-MI group revealed a pre-ischemic peripheral monocytosis as compared to the NC-MI group as expressed by the percentage of monocytes (41.6±2.1% vs. 33.6±1.6% of total leukocytes, *P* = 0.009, Fig 5A), which was normalized three weeks after MI-R. Furthermore, the HC-MI group showed a higher percentage pro-inflammatory Ly-6C^hi^ monocytes of the total monocyte population in peripheral blood prior to MI-R (40.8±2.7% vs. 30.6±2.2% of total monocytes, *P* = 0.01, Fig 5B), which was normalized three weeks after MI-R as well. These results suggest a loss of total and in particular Ly-6C^hi^ monocytes from peripheral blood of HC-MI mice due to MI-R. In contrast, eight weeks after MI-R a non-significant decreased number of infiltrated CD45^+^ leukocytes was observed in the infarct area of the HC-MI group as compared to the NC-MI group (4.7±0.4 vs. 7.0±2.0 cells per 0.25mm^2^, *P* = 0.27, Fig 5C) whereas the number of macrophages was significantly reduced (7.9±1.8 vs. 20.7±5.4 cells per 0.25mm^2^, *P* = 0.049, Fig 5D).

## Discussion

Key findings of the present study are that diet-induced hypercholesterolemia in APOE*3-Leiden mice caused a pre-ischemic peripheral monocytosis, in particular of the Ly-6C^hi^ pro-inflammatory monocytes, and impaired intrinsic LV function eight weeks after acute MI-R. Paradoxically, this was accompanied by a reduced infarct size and a suppressed accumulation of infiltrated inflammatory cells in the ischemic-reperfused myocardium after eight weeks. To our knowledge this study is the first to extend follow-up after MI-R to a period of eight weeks regarding the effects of hypercholesterolemia. Furthermore, this experimental model using hypercholesterolemic APOE*3-Leiden mice exposed to MI-R seems suitable to study novel cardioprotective therapies in a more clinically relevant animal model aiming for improved translation into the complex clinical reality of reperfused STEMI patients in the end as was also suggested in a recent ESC position paper [18].

### Impaired cardiac function as a result of hypercholesterolemia

In the present study, hypercholesterolemia resulted in impaired intrinsic LV function eight weeks after MI-R as reflected by a reduced left ventricular ESP, dP/dt_MAX_, and dP/dt_MIN_., whereas no differences of LV dimensions were observed. These results are in line with previous studies reporting that unreperfused MI in HC animals results in depressed LV function in rabbits [38] and exacerbated LV diastolic dysfunction in rats [39] at least eight weeks after ischemia. Hypercholesterolemia itself was suggested to cause cardiomyopathy by formation of myocardial cholesterol deposits. Shifting of ATP production from glucose to free fatty acids increased free radicals which resulted in myocardial injury [40]. A subsequent resulting reduced hemodynamic performance caused by hypercholesterolemia may lead to a decreased metabolic demand. This is proposed to confer a cardioprotective state [9,41] that may lead to improved post-ischemic functional recovery in rabbit hearts. Hypercholesterolemia may therefore increase myocardial tolerance against ischemia [10].

### Reduction of infarct size

The reduced infarct size accompanied by a preserved LV wall thickness of the infarcted myocardium in the HC animals may have been caused by the abovementioned reduced myocardial metabolic demand resulting in increased tolerance against myocardial ischemia. Another explanation could be a difference in the post-ischemic inflammatory response since we showed a concomitantly reduced influx of leukocytes and macrophages in the post-ischemic myocardium. This is supported by previous studies in humans and rats which reported a positive correlation between infiltrated inflammatory cells and infarct size [42,43].

In accordance to our long-term results eight weeks after MI-R, MI-R in HC mice also resulted in smaller infarcts two hours after onset of reperfusion and was concluded to provide cardioprotection in mice [17]. On the other hand, experimental studies using HC MI-R models with a short-term follow-up (24 hours or less) reported an increased infarct size in rabbits [15], as well as increased myeloperoxidase expression in rats [13] and rabbits [12] and increased cardiomyocyte apoptosis [11]. In addition permanent ischemia in hypercholesterolemic rabbits [38] or rats [39] caused no difference in infarct size eight weeks after MI, endorsing conflicting results from current literature.

### Pre-ischemic monocytosis followed by reduced integrated inflammatory cells in the ischemic-reperfused myocardium

A reduced influx of leukocytes and macrophages was observed in the ischemic-reperfused myocardium of HC mice, preceded by a pre-ischemic hypercholesterolemia-associated peripheral monocytosis. Hypercholesterolemia has been reported to cause a peripheral monocytosis with regard to the Ly-6C^hi^ subset in HC mice [44]. In addition, the inflammatory response after MI-R is more complex as compared to unreperfused MI, since reperfusion itself induces a pathophysiological process of reperfusion injury [45]. The pro-inflammatory state resulting from this monocytosis could affect the inflammatory response following MI-R mediated by cytokines and chemokines, since Ly-6C^hi^ monocytes are involved in the initial inflammatory response following ischemia. Previous studies demonstrated that within the first hours after MI, monocytes and their lineage descendant macrophages infiltrate the infarcted myocardium resulting in the release of cytokines and growth factors, phagocytosis of debris, clearance of apoptotic cells, and the release of proteases [46,47]. Especially the pro-inflammatory Ly-6C^hi^ monocyte subset is known to promote digestion of infarcted tissue and clearance of necrotic debris [48]. After myocardial ischemia, a baseline Ly-6C^hi^ monocytosis could therefore favorably affect the subsequent Ly-6C^lo^-mediated reparative phase accelerating repair thereby limiting tissue damage on the long-term. Conversely, atherosclerosis-related leukocytosis was found to disturb the acute post-ischemic healing process [31]. Following permanent ischemia, Ly-6C^hi^ monocytosis has also been reported to disturb infarct healing and enhance left ventricular remodeling after three weeks [49]. This could probably be explained by a clearly different post-ischemic inflammatory response after unreperfused MI compared to the more complex inflammatory response after MI-R [46].

In summary, hypercholesterolemia in an *in vivo* APOE*3-Leiden mouse model causes a pre-ischemic peripheral monocytosis and impaired systolic and diastolic cardiac function eight weeks after myocardial ischemia-reperfusion injury. This is accompanied however with a decreased myocardial infarct size and a reduced accumulation of inflammatory cells in the ischemic-reperfused myocardium.

## Supporting information

S1 Fig Timeline of the complete study protocol. A schematic overview of the complete study protocol shown as a timeline.
(TIF)

## Abbreviations

APOE*3-Leiden: apolipoprotein E3-Leiden
CO: cardiac output
EF: ejection fraction
FACS: fluorescence-activated cell sorting
HC: hypercholesterolemic
LAD: left anterior descending
LV: left ventricular
EDP: end-diastolic pressure
EDV: end-diastolic volume
ESP: end-systolic pressure
ESV: end-systolic volume
MI: myocardial infarction
MI-R: myocardial ischemia-reperfusion
NC: normocholesterolemic
TC: total cholesterol
TG: triglycerides
