# Supplementary figures and images for: Hypercholesterolemia affects cardiac function, infarct size and inflammation in APOE*3-Leiden mice following myocardial ischemia-reperfusion injury
Source: PLoS One. 2019 Jun 14;14(6):e0217582. doi: 10.1371/journal.pone.0217582 (PMC6570022; doi:10.1371/journal.pone.0217582)

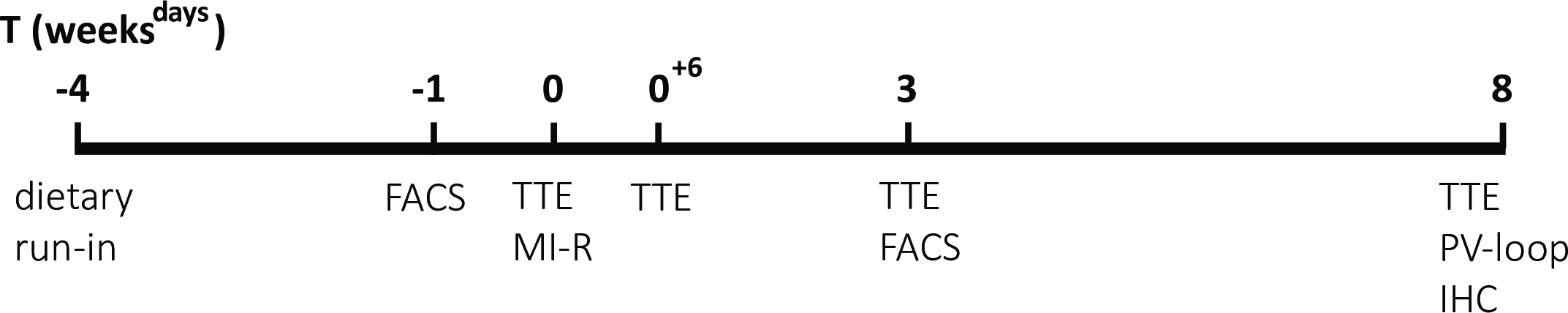

Supplement: S1 Fig — A schematic overview of the complete study protocol shown as a timeline. (TIF) [file pone.0217582.s001.tif]
